# Tumor expression, plasma levels and genetic polymorphisms of the coagulation inhibitor TFPI are associated with clinicopathological parameters and survival in breast cancer, in contrast to the coagulation initiator TF

**DOI:** 10.1186/s13058-015-0548-5

**Published:** 2015-03-26

**Authors:** Mari Tinholt, Hans Kristian Moen Vollan, Kristine Kleivi Sahlberg, Sandra Jernström, Fatemeh Kaveh, Ole Christian Lingjærde, Rolf Kåresen, Torill Sauer, Vessela Kristensen, Anne-Lise Børresen-Dale, Per Morten Sandset, Nina Iversen

**Affiliations:** Department of Medical Genetics, Oslo University Hospital and University of Oslo, BOX 4956, Nydalen, Oslo, N-0424 Norway; Department of Haematology and Research Institute of Internal Medicine, Oslo University Hospital, Oslo, Norway; Institute of Clinical Medicine, University of Oslo, Oslo, Norway; Department of Genetics, Institute for Cancer Research, Oslo University Hospital Radiumhospitalet, Oslo, Norway; The K.G. Jebsen Center for Breast Cancer Research, Institute of Clinical Medicine, Faculty of Medicine, University of Oslo, Oslo, Norway; Department of Oncology, Division of Surgery, Transplantation and Cancer Medicine, Oslo University Hospital Radiumhospitalet, Oslo, Norway; Department of Research, Vestre Viken, Drammen, Norway; Biomedical Informatics Research Group, Department of Informatics, University of Oslo, Oslo, Norway; Department of Breast and Endocrine Surgery, Oslo University Hospital, Oslo, Norway; Department of Pathology, Akershus University Hospital, Lørenskog, Norway; Department of Clinical Molecular Biology (EpiGen), Akershus University Hospital, Lørenskog, Norway

## Abstract

**Introduction:**

Hypercoagulability in malignancy increases the risk of thrombosis, but is also involved in cancer progression. Experimental studies suggest that tissue factor (TF) and tissue factor pathway inhibitor (TFPI) are involved in cancer biology as a tumor- promoter and suppressor, respectively, but the clinical significance is less clear. Here, we aimed to investigate the clinical relevance of TF and TFPI genetic and phenotypic diversity in breast cancer.

**Methods:**

The relationship between tumor messenger RNA (mRNA) expression and plasma levels of TF and TFPI (α and β), tagging single nucleotide polymorphisms (tagSNPs) in *F3* (*TF*) (n = 6) and *TFPI* (n = 18), and clinicopathological characteristics and molecular tumor subtypes were explored in 152 treatment naive breast cancer patients. The effect of tumor expressed TF and TFPIα and TFPIβ on survival was investigated in a merged breast cancer dataset of 1881 patients.

**Results:**

Progesterone receptor negative patients had higher mRNA expression of total TFPI (α + β) (*P* = 0.021) and TFPIβ (*P* = 0.014) in tumors. TF mRNA expression was decreased in grade 3 tumors (*P* = 0.003). In plasma, total TFPI levels were decreased in patients with larger tumors (*P* = 0.013). SNP haplotypes of *TFPI*, but not *TF*, were associated with specific clinicopathological characteristics like tumor size (odds ratio (OR) 3.14, *P* = 0.004), triple negativity (OR 2.4, *P* = 0.004), lymph node spread (OR 3.34, *P* = 0.006), and basal-like (OR 2.3, *P* = 0.011) and luminal B (OR 3.5, *P* = 0.005) molecular tumor subtypes. Increased expression levels of TFPIα and TFPIβ in breast tumors were associated with better outcome in all tumor subtypes combined (*P* = 0.007 and *P =* 0.005) and in multiple subgroups, including lymph node positive subjects (*P* = 0.006 and *P* = 0.034).

**Conclusions:**

This study indicates that genetic and phenotypic variation of both TFPIα and TFPIβ, more than TF, are markers of cancer progression. Together with the previously demonstrated tumor suppressor effects of TFPI, the beneficial effect of tumor expressed TFPI on survival, renders TFPI as a potential anticancer agent, and the clinical significance of TFPI in cancer deserves further investigation.

**Electronic supplementary material:**

The online version of this article (doi:10.1186/s13058-015-0548-5) contains supplementary material, which is available to authorized users.

## Introduction

There is now convincing evidence of a relationship between cancer and hemostasis. An oncogene driven thrombophilic environment frequently arises in cancer and involves the capacity of tumor- and stimulated host stroma cells to express and release clotting factors, inflammatory cytokines, proangiogenic factors, and procoagulant microparticles (MPs) [1]. One obvious manifestation of cancer-associated coagulopathy is the increased risk of venous thrombosis (VT) among cancer patients [2-4], however, the procoagulant state may also promote tumor progression [5]. Full-length tissue factor (*TF*) [GenBank: NM_001993] is the most extensively studied coagulation factor in cancer, and its activity is regulated by TF pathway inhibitor (TFPI). Expression of both TF and TFPI has been detected in tissues and cell lines of several human cancers including breast cancer [6-10], suggesting a role in cancer biology. TF is known to be a trigger of angiogenesis, proliferation, migration, and invasion, and to prevent apoptosis [11-14]. These effects may either be coagulation dependent, indirectly through fibrin formation and platelet activation, or through coagulation independent signaling via factor VIIa (FVIIa) and activation of protease activated receptor 2 (PAR-2), enhanced by β1 integrin [5,12].

Full-length *TFPI* (*TFPI*α) [GenBank: NM_006287] consists of three Kunitz-type inhibitor domains and a positively charged carboxy-terminal (C-terminal) end, while the alternatively spliced *TFPI*β [GenBank: NM_001032281] lacks the third Kunitz domain and has a unique C-terminal that directs binding to a glycosylphosphatidylinisotol (GPI) anchor on the cell surface [15]. There is growing evidence for non-hemostatic tumor-suppressive activities of TFPI. Studies of endothelial cell cultures have shown that recombinant TFPIα induces apoptosis [16], inhibits proliferation via the very low density lipoprotein receptor [17], and show anti-angiogenic and anti-migratory properties [18,19]. In addition, manipulation of their expression revealed that both the TFPIα and the TFPIβ isoform have tumor-suppressive features in breast cancer cells, similar to that observed in endothelial cells [20,21]. Supporting the *in vitro* observations, *in vivo* studies have demonstrated that both circulating recombinant TFPI and TFPI-expressing tumor cells significantly attenuated tumor growth [13,22] and lung metastasis in mice [13,23]. A transgenic murine model of TFPI overexpression suggested that the C-terminal end of TFPIα caused impaired angiogenesis by inhibition of phosphorylation of vascular endothelial growth factor receptor 2 [19]. A few *TFPI* and *TF* (*F3*) single nucleotide polymorphisms (SNPs) have been reported and assigned a possible, but not definite role in modifying transcription or plasma levels of TFPI and TF [24-30].

Breast cancer is a highly heterogeneous disease and several subgroups exist that differ in prognosis and management options. Three main tumor subtypes may be determined using traditional immunohistochemistry (IHC); hormone receptor (HR) positive (i.e., estrogen receptor (ER) and progesterone receptor (PR) positive), human epidermal growth factor receptor 2 (HER2) positive, and triple negative tumors. In addition, whole-genome gene expression profiling has enabled categorization of tumors into the intrinsic molecular subtypes luminal A, luminal B, HER2-enriched, basal-like and normal-like [31].

In the present study, we aimed to investigate the clinical relevance of TFPI and TF in breast cancer. The inter-relationships between TFPI and TF tagSNPs, tumor mRNA expression and plasma levels, and their association with breast cancer subtypes and survival were explored in either 152 treatment naive breast cancer patients or a merged 1881-sample breast tumor data set.

## Materials and methods

### Case subjects

The study comprised 152 primary operable (cT1-cT2) female breast cancer patients (Table 1) enrolled between June 2008 and August 2010 at the Oslo University Hospital Ullevål, Oslo, and the Akershus University Hospital, Nordbyhagen, Norway. All subjects were of Scandinavian descent (mainly Norwegian, some Swedish or Danish). The cases were included at the time of primary surgery (mastectomy or lumpectomy), without receiving any pre-operative treatment. Blood samples were drawn immediately before surgery and tumor tissue was fresh frozen in liquid nitrogen and stored at −80°C after macroscopic evaluation of the surgical specimen by an experienced breast pathologist. None of the subjects were pregnant or received any anticoagulant- or hormone replacement therapy. The age average at the time of surgery and blood sampling was 56.0 (standard deviation (SD) 12.4) years. The Norwegian southeastern Regional Committee for Medical and Health Research Ethics approved the study protocols (approval number 1.2006.1607, amendment 1.2007.1125 for Ullevål patients and 429–04148 for Akershus patients) and all included women gave their written informed consent to participate.

**Table 1:** **Clinicopathological characteristics of the 152 breast cancer patients**

**Characteristic**	**Number (%)**
**Gender**	
Female	152 (100)
Male	0 (0)
**Age**	
≤55	80 (52.6)
>55	72 (47.4)
Mean (±SD)	56.0 (12.4)
**Tumor size (T-status)**	
T1 (0–20 mm)	77 (50.7)
T2 (20–50 mm)	68 (44.7)
T3 (>50 mm)	7 (4.6)
(T2-T3)	(75 (49.3))
**Grade**	
G1 (well differentiated)	21 (13.8)
G2 (moderately differentiated)	53 (34.9)
(G1-G2)	(74 (48.7))
G3 (poorly differentiated)	78 (51.3)
**Estrogen receptor (ER) status**	
Negative	35 (23.0)
Positive	117 (77.0)
**Progesterone receptor (PR) status**	
Negative	51 (33.6)
Positive	101 (66.4)
**Human epidermal growth factor receptor 2 (HER2) status**	
Negative	137 (90.1)
Positive	15 (9.9)
**Hormone receptor (HR) negative status (ER/PR negative)**	
Yes	34 (22.4)
No	118 (77.6)
**Triple negative (TN) status (ER/PR/HER2 negative)**	
Yes	29 (19.1)
No	123 (80.9)
**Lymph node (N) status**	
N0 (0 lymph nodes)	98 (64.5)
N1 (1–3 lymph nodes)	40 (26.3)
N2 (4–9 lymph nodes)	11 (7.2)
N3 (≥10 lymph nodes)	3 (2.0)
(N1-N2-N3)^a^	(54 (35.5))
**Molecular tumor subtypes (PAM50 signatures)**	
Basal	25 (16.7)
HER2 enriched	15 (10.0)
Luminal A	63 (42.0)
Luminal B	39 (26.0)
Normal-like	8 (5.3)

^a^Lymph node positive group.

### Clinicopathological characteristics

Clinicopathological data were retrieved from pathology reviews, and the following clinicopathological characteristics were included in the study; tumor size (T-status), lymph node (N) status, tumor grade (well/moderately/poorly differentiated), ER status, PR status, and HER2 status (Table 1). T-status, N-status and tumor grade was specified according to national (NBCG, [32] guidelines and World Health Organization (WHO) recommendations. ER and PR status of the tumors were determined by IHC, and tumor cell nuclei were scored according to pathology guidelines. HER2 status was determined by IHC and/or by silver enhancement in situ hybridization (SISH) (Roche, Dual SISH HER-2) where a HER2 gene/centrosome 17 (CEP17) ratio of > 2.2 defined HER2 positivity.

### Tumor preparation

Tumor tissue from each patient was cut into three pieces and two frozen sections were taken, Hematoxylin and Eosin (HE) stained, and evaluated for the presence of tumor cells. Afterwards, the three tumor pieces were combined and cut into small pieces. DNA and RNA isolation were thereafter performed from the mixed tumor tissue.

### RNA isolation and TFPI and TF mRNA expression in tumor

Total RNA was isolated from the breast tumors with Trizol and quality controlled using the Agilent 2100 Bioanalyzer. 100 ng total RNA was applied to the SurePrint G3 Human GE 8x60K one-color microarrays (Agilent Technologies, Santa Clara, CA, USA). Scanning was performed with Agilent Scanner G2565A, using AgilentG3_GX_1Color as profile. Signals were extracted using FE v.10.7.3.1 and protocol GE1_107_Sep09 (Agilent Technologies). Probe-values were log2-transformed, and samples were quantile normalized and hospital-adjusted by subtracting from each probe value the median probe value among samples from the same hospital. In the present study, only expression ratio results obtained with *TFPI* and *TF* (*F3*) probes were used for further analysis (Additional file 1: Table S1). The three *TFPI* probes present on the microarray were specific for 1) the TFPIα isoform, 2) the TFPIβ isoform, and 3) total TFPI expression (TFPIα + TFPIβ). Four SNPs were observed in these probe regions (*TFPI* rs79927400, *TFPI* rs187580582, *TF* rs3917635, and *TF* rs191529173), however, according to the 1000 genome project [33] all were low frequent with a minor allele frequency (MAF) ≤1%. A deletion (rs71653267) in the *TF* probe has been reported (dbSNP build 138), however, with unknown significance and frequency. No repetitive DNA elements were found within any of the probe regions.

The microarray data have been submitted to the Gene Expression Omnibus (GEO) database with accession number GSE58215.

### PAM50 subtyping

The prediction analysis of microarray 50 (PAM50) subtype algorithm described by Parker *et al*. [34] was used to assign a subtype label to each sample. In short, a combined centroid was defined as a weighted average of the centroids for ER-negative and ER-positive samples, and each sample was centered by aligning the combined centroid with the centroid of the training data set provided in [34], achieved by subtracting the combined centroid from the expression vector of each sample and then adding the centroid of the training data set. Subtype labels (luminal A, luminal B, basal, HER2 enriched, and normal-like) were assigned by calculating the Spearman correlation between the expression vector of each individual sample and each of the five PAM50 centroids. The subtype with the highest correlation was selected.

### DNA isolation from whole-blood and tumor material

DNA from whole blood was either isolated on the BioRobot Universal with the QIAamp DNA Blood BioRobot MDx Kit (Qiagen, Hilden, Germany) and eluted in Qiagen buffer AE (10 mM Tris-Cl 0.5 mM EDTA; pH 9.0), or with the Gentra Autopure LS machine using the Puregene Genomic DNA purification Kit (Gentra Systems, Minneapolis, MN 55441 USA), or manually using the MasterPure TM DNA Purification Kit for Blood Version II (Epicentre® Biotechnologies, Madison, WI, USA).

Fresh frozen tumor tissue was cut with scalpel, and one piece was used for DNA isolation using the Maxwell® 16 instrument (Promega, Madison, WI, USA) and the Maxwell® 16 tissue DNA Purification Kit (Promega). DNA was isolated according to the manufacturer’s protocol. The isolation procedure is automated, starting with sample lysis and tissue homogenization, followed by bead isolation of DNA, and finally the washing steps. The DNA was eluted in TE buffer (pH 8.5). DNA was stored at −20°C. DNA concentration and quality were measured using NanoDrop® ND-1000 (NanoDrop Technologies, Wilmington, DE, USA).

### SNP selection, genotyping, and quality control

SNPs in the *TFPI* and *TF* gene regions were selected using a SNP tagging approach. The tagSNP selection was performed using the Tagger program [35] implemented in Haploview v. 4.2. and genotype data from the Caucasian population (Utah residents with ancestry from Northern and Western Europe) from the HapMap project release 27, phase III on NCBI B36 assembly, dbSNPb126. Using a MAF criterion of ≥5% and pairwise r^2^ ≥ 0.8 as a cut-off for proxies, sixteen tagSNPs in the *TFPI* gene and four tagSNPs in the *TF* gene were selected for genotyping. In addition, three SNPs in *TFPI* (rs5940, rs10931292 and rs10176633) and two SNPs in *TF* (rs958587 and rs3917643) with a previously suggested expression regulatory function were included. Thus the final SNP selection consisted of nineteen *TFPI* SNPs and six *TF* SNPs. However, one of the *TFPI* SNPs failed to design (rs8176500), leaving eighteen *TFPI* SNPs for genotyping.

Germline SNPs were genotyped in whole blood (fourteen *TFPI* SNPs and six *TF* SNPs) or tumor tissue (four *TFPI* SNPs) using the iPLEX Gold massarray platform (Sequenom) at the Centre for Integrative Genetics, Norwegian University of Life Sciences, Ås, Norway, or the Affymetrix**®** Genome**-**Wide Human SNP Array 6.0 at AROS Applied Biotechnology AS, Aarhus, Denmark, respectively. Due to the tumor genome being prone to mutational and copy number changes, including loss of heterozygosity (LOH), the tumor-derived SNP genotypes may occasionally deviate from the germline genotypes. Therefore, the tumor-derived SNP data were processed to deduce germline SNPs. With access to blood-derived germline genotype data for all patients on a set of 22 SNPs (rs8176548, rs3917615, rs696619, rs2227607, rs2227589, rs12488200, rs9332542, rs9332618, rs6012, rs4524, rs491098, rs421766, rs4149762, rs4149674, rs473598, rs3211752, rs2227426, rs2070022, rs153311, rs2227750, rs37246, rs2070852) present on the Human SNP Array 6.0, we verified a convergence of 93.8% between the deduced germline SNPs (from tumor) and the true germline SNPs (from blood). Due to this fairly high genotype agreement, we concluded that the tumor-derived SNPs included in this study (n = 4, indicated in Additional file 2: Table S2) could be considered as germline SNPs.

All SNPs were in Hardy-Weinberg equilibrium (significance threshold *P* < 0.001). Genotyping was successful for ≥50% of the SNPs in all individuals. Three *TFPI* SNPs had genotyping call rates <97% (rs2041778, rs10931292 and rs8176508), and one *TFPI* SNP was monomorphic (rs10176633). After removal of these four SNPs, fourteen *TFPI* SNPs (Additional file 2: Table S2 and Additional file 3: Figure S1) and six *TF* SNPs (Additional file 2: Table S2) remained for further analysis.

### Blood sampling and TFPI/TF protein levels in plasma

Venous blood samples were collected in Vacutainer® vacuum tubes (Becton-Dickinson, Plymouth, UK) containing 0.5 mL buffered sodium citrate (0.129 mol/L). Whole blood was centrifuged for 15 min at 2000 g at room temperature within 1 hour to prepare platelet poor plasma, and aliquots were stored at −70°C until analyzed. TFPI protein levels in plasma were determined using the commercial Free and Total TFPI ELISA kits (Asserachrom®, Diagnostica Stago, Asnière, France), while the Zymutest Tissue Factor full-length (RK035A, Hyphen BioMed, Neuville-Sur-Oise, France) was used to measure TF protein. The manufacturers’ recommended protocols were followed.

### Statistical methods

Statistical analyzes were performed using SPSS (version 21.0; SPSS Inc., Chicago, IL, USA), PLINK v.1.07 [36], and R 3.1.0 [37]. Associations between continuous-valued expression levels were assessed with Pearson correlation or Spearman rank correlation. The relation between SNP genotypes and expression levels was investigated by linear regression. Genotypes were treated as ordinal variables (0, 1 or 2 copies of the minor allele), thus assuming potential allele-dosage effects to be linear. Regression coefficients are denoted B and the correlation coefficients are denoted r.

For associations between expression levels and clinicopathological characteristics, heatmaps were constructed in R. Missing value imputation of the gene expression data was performed with LSimpute [38] using the row mean method. Two-group comparisons of expression levels were performed using the t-test or the Mann–Whitney U test as appropriate. One-way ANOVA or Kruskal-Wallis test was used for multi-group comparisons. Associations between SNPs and binary clinicopathological characteristics were examined using logistic regression under the additive risk model, with the clinicopathological variable(s) as the dependent variable and genotype(s) (coded 0, 1, 2 for each extra risk allele) as the categorical independent variable. Risk alleles were defined as the alleles being more prevalent among the non-reference category, thus consequently obtaining positive odds ratios (ORs >1). ORs with corresponding 95% confidence intervals (CIs) were reported.

Independence between SNPs was tested by conditional analysis in PLINK, where the allelic dosage for a given SNP was added as a covariate in a logistic regression model. Pairwise SNP dependencies exist if individual effects disappear when conditioned on the other SNP. The expectation–maximization (EM) algorithm was used to estimate haplotype frequencies, and haplotype-based association analysis was conducted using logistic regression (additive model) for associations with clinicopathological variables and linear regression for associations with expression levels. Haploview v. 4.2 was used for creating linkage disequilibrium (LD) plots. False discovery rate (FDR, [39]) was used to account for multiple testing, and tests with *P* < 0.05 and FDR < 0.20 were reported.

### Bioinformatic analysis using the Gene expression based Outcome for Breast cancer Online (GOBO) database

The GOBO database is a web-based tool that enables a variety of analyzes of gene expression data in a merged 1881-sample breast tumor data set of 11 different publicly available datasets, all generated on Affymetrix U133A microarrays [40]. Four TFPI specific probe sets (one for total TFPI (α + β), two for TFPIα, one for TFPIβ) and one probe set for *TF* gene expression were identified (Additional file 4: Table S3).

Using the Gene Set Analysis application, the *TFPI* and *TF* gene expressions were dichotomized to levels above the median (high expression) or below the median (low expression) before Kaplan-Meier plots were created and univariate analyzes (log-rank) were performed to predict 10-year censored overall and relapse free survival. This was conducted in the complete 1881-sample merged clinical data set (hereafter termed “all tumors”) as well as in clinical subgroups. Multivariate Cox proportional hazards regression analyzes were carried out to account for possible survival effects of the following covariates; tumor size, age, histological tumor grade, lymph node status and ER-status. In addition, GOBO was used to assess the distribution of TFPI and TF gene expressions across clinical subgroups.

## Results

### TFPI and TF expression in breast cancer subtypes

**Figure 1:**
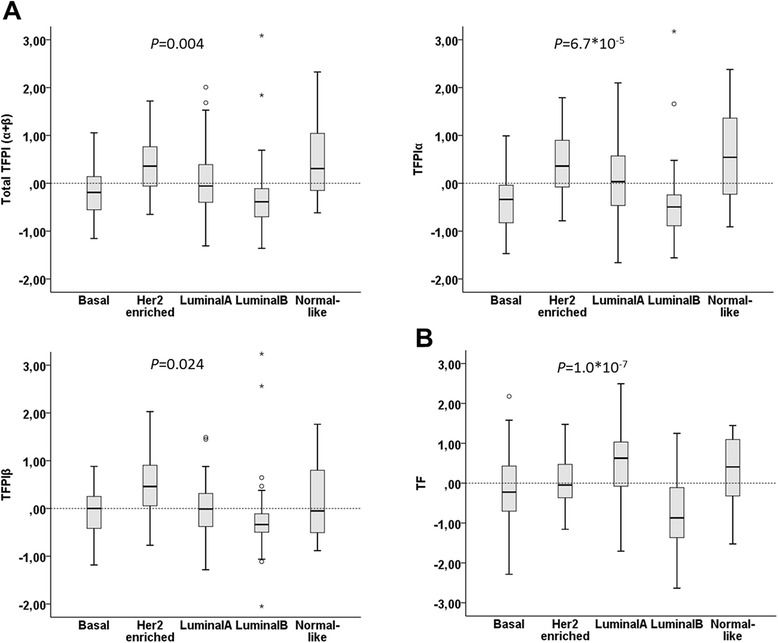
**Tissue factor pathway inhibitor (TFPI) and tissue factor (TF) mRNA expression across molecular breast cancer subtypes.** Box and whisker plot showing the distribution of log2-transformed total TFPI (α + β), TFPIα, and TFPIβ **(A)**, and *TF* tumor mRNA expression **(B)** across the following intrinsic molecular subtypes of 150 of the 152 breast cancer patients; basal (n = 25), human epidermal growth factor 2 (HER2)-enriched (n = 15), luminal A (n = 63), luminal B (n = 39) and normal-like (n = 8). *P*-values for multi-group comparison are indicated.

**Figure 2:**
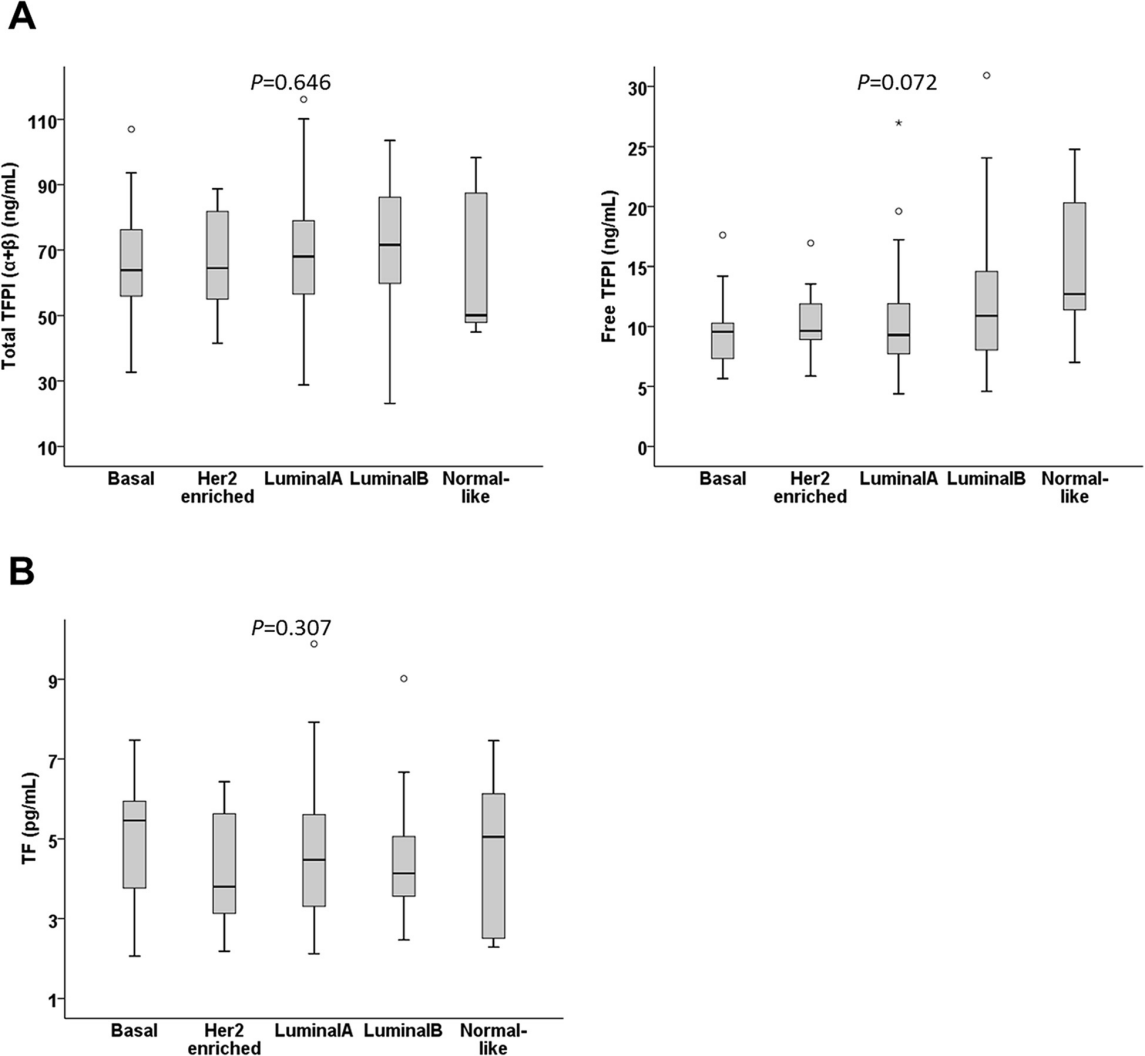
**Tissue factor pathway inhibitor (TFPI) and tissue factor (TF) plasma levels across molecular breast cancer subtypes.** Box and whisker plot showing the distribution of plasma levels of total TFPI and free TFPI **(A)** and TF **(B)** across the following intrinsic molecular subtypes of 148 of the 152 breast cancer patients; basal (n = 24), human epidermal growth factor 2 (HER2) HER2-enriched (n = 15), luminal A (n = 62), luminal B (n = 39) and normal-like (n = 8). *P*-values for multi-group comparison are indicated.

First, we investigated the clinical relevance of TFPI or TF mRNA expression in breast tumors and TFPI or TF plasma concentrations. Patients were stratified according to clinicopathological tumor characteristics as defined in Table 2, with median expression levels for each group. Significant differences in mRNA expression or protein levels are illustrated by heatmaps (Additional file 5: Figure S2). TFPI or TF mRNA expression in breast tumors and plasma concentrations were then compared between the five molecular tumor subtypes (Figures 1 and 2, respectively).

**Table 2:** **TFPI and TF tumor mRNA expression across clinicopathological breast cancer subtypes**

		**mRNA expression (tumor)**								**Protein levels (plasma)**					
**Characteristic**	**Groups**	**Total TFPI (α + β)**	***P***	**TFPIα**	***P***	**TFPIβ**	***P***	**TF**	***P***	**Total TFPI**	***P***	**Free TFPI**	***P***	**TF**	***P***
T-status	T1	−0.146	0.054	−0.135	0.257	−0.084	0.201	−0.023	0.652	72.01	**0.013**	10.82	0.997	4.14	0.125
	T2-T3	0.085		0.018		0.060		0.054		65.02		10.82		4.66	
Grade	G1-G2	−0.022	0.850	−0.005	0.424	−0.033	0.743	0.271	**0.003**	71.04	0.082	10.66	0.682	4.63	0.557
	G3	−0.045		−0.113		0.004		−0.229		66.12		10.97		4.14	
N-status	Negative	−0.109	0.091	−0.136	0.127	−0.082	0.104	0.005	0.881	69.93	0.183	10.77	0.869	4.95	0.282
	Positive	0.104		0.078		0.110		0.032		66.00		10.90		4.14	
ER status	Positive	−0.067	0.317	−0.082	0.557	−0.056	0.183	0.001	0.784	69.42	0.240	10.91	0.671	4.42	0.409
PR status	Negative	0.076		0.011		0.123		0.057		65.44		10.52		5.28	
	Positive	−0.131	**0.021**	−0.145	0.075	−0.112	**0.014**	0.085	0.244	69.81	0.195	11.19	0.175	4.32	0.246
HER2-status	Negative	0.161		0.108		0.182		−0.127		65.92		10.08		5.04	
	Negative	−0.072	0.054	−0.101	0.073	−0.041	0.154	0.004	0.731	68.45	0.893	10.68	0.287	4.47	0.428
	Positive	0.313		0.301		0.228		0.103		69.09		12.05		4.78	
HR status	Yes	0.076	0.326	0.007	0.587	0.114	0.221	0.016	0.991	64.78	0.161	10.41	0.568	5.26	0.470
	No	−0.066		−0.080		−0.052		0.014		69.57		10.94		4.47	
Triple-negative status	Yes	−0.051	0.886	−0.110	0.718	0.041	0.635	−0.158	0.326	63.21	0.072	10.06	0.345	5.23	0.969
	No	−0.029		−0.048		−0.027		0.055		69.73		10.99		4.57	

Median values for TFPI and TF mRNA expression in tumors and protein levels in plasma according to clinically defined groups. Corresponding *P*-values (unadjusted) are shown. Significant *P*-values in bold. TFPI, tissue factor pathway inhibitor; TF, tissue factor; HER2, human epidermal growth factor receptor 2.Abbreviations: T, tumor; G, grade; N, node; ER, estrogen receptor; PR, progesterone receptor; HR, hormone receptor.

Total TFPI (α + β) mRNA expression was increased in patients with PR-negative tumors (*P* = 0.021), and in patients with HER2-positive tumors, larger tumor sizes, and positive lymph nodes, although power was lacking to achieve statistical significance. Expression of the TFPIα isoform was higher in PR-negative and HER2-positive patients, whereas TFPIβ was significantly higher only in PR-negative patients (*P* = 0.014). No differences in TFPI mRNA expression were observed between the stratified groups for grade, ER status, HR status, or triple-negative status (Table 2). Moreover, total TFPI (α + β), TFPIα and TFPIβ expression levels were differentially distributed among the five molecular breast cancer subtypes; with higher expression in the HER2-enriched and the normal-like group, and lower in luminal B tumors (Figure 1A). These observations were mainly supported by equivalent analyses of the merged breast cancer dataset in GOBO (Additional file 6: Figure S3).

TF mRNA expression was significantly decreased in patients with grade-3 tumors compared with patients with grade 1 or 2 (*P* = 0.003) (Table 2), and this was validated by gene expression pattern analysis of the merged breast cancer dataset (Additional file 7: Figure S4). When stratified by molecular subtypes, *TF* mRNA expression was higher in luminal A and the normal-like tumors, and lowest in luminal B tumors (Figure 1B). Similar results were obtained using the merged breast cancer dataset (Additional file 6: Figure S3).

In plasma, antigen levels of free TFPI and TF were similar between all stratified clinical groups, whereas total TFPI was decreased in patients with larger tumors (*P* = 0.013), and patients with grade-3 or triple-negative tumors (not significant) (Table 2). Total TFPI or free TFPI (Figure 2A), and TF plasma levels (Figure 2B) were not significantly different across molecular subtypes.

As expected, the total TFPI (α + β), TFPIα and TFPIβ mRNA expression levels in breast tumors were highly correlated (*r* = 0.82 to 0.91, *P* <0.001), and did also correlate significantly to TF expression (*r* = 0.37-0.50, *P* <0.001). In plasma, total TFPI protein levels correlated significantly to free TFPI (*r* = 0.36, *P* <0.001) and TF (*ρ* = 0.25, *P* = 0.002), whereas no correlation was found between free TFPI and TF. No significant correlation between protein levels (in plasma) and mRNA expression (in tumors) was found for TFPI or TF.

### Association between SNPs in TFPI and TF and breast cancer subtypes

Next, we evaluated if *TFPI* or *TF* SNPs (Additional file 2: Table S2) were associated with any clinicopathological characteristics and molecular tumor subtypes. Four *TFPI* SNPs (rs8176541, rs3213739, rs8176479, and rs2192824) were associated with triple-negative status (Table 3) as well as ER status and HR status (data not shown). Strong pairwise LD existed (Additional file 3: Figure S1), and conditional association analysis supported SNP dependency (data not shown). Haplotype-based analysis showed that the common G-G-C-T haplotype (frequency 0.41) formed by the four SNPs (rs8176541-rs3213739-rs8176479-rs2192824) showed an OR of 2.4 (*P* = 0.004) in triple-negative patients (Table 3).

**Table 3:** **Significant association between** ***TFPI*** **single nucleotide polymorphisms (SNPs) and clinicopathological characteristics and molecular subtypes**

**Characteristic**	**SNP**	**Risk allele**	**Odds ratio**	**95% CI**	***P***	**False discovery rate**
**T status**						
T1			Reference	Reference	Reference	Reference
T2 to T3	rs10153820	A	3.14	1.44, 6.86	0.004	0.056
**TN status** (ER-/PR-/HER2-negative)						
No			Reference	Reference	Reference	Reference
Yes	rs8176541^a^	G	2.62	1.11, 5.35	0.026	0.092
	rs3213739^a^	G	2.58	1.34, 4.99	0.005	0.033
	rs8176479^a^	C	3.10	1.24, 7.72	0.015	0.071
	rs2192824^a^	T	2.44	1.39, 4.93	0.002	0.033
**N status**						
Positive			Reference	Reference	Reference	Reference
Negative	rs10179730	G	3.34	1.42, 7.89	0.006	0.083
**Basal tumor subtype**						
Non-basal			Reference	Reference	Reference	Reference
Basal	rs3213739^a^	G	2.23	1.15, 4.34	0.018	0.107
	rs8176479^a^	C	2.79	1.12, 6.96	0.028	0.107
	rs2192824^a^	T	2.41	1.24, 4.65	0.009	0.107
	rs10187622^a^	C	5.20	1.17, 23.20	0.031	0.107
**Luminal B tumor subtype**						
Non-luminal B			Reference	Reference	Reference	Reference
Luminal B	rs16829086^a^	T	2.09	1.03, 4.25	0.041	0.191
	rs10179730^a^	G	3.53	1.47, 8.46	0.005	0.066
	rs10187622^a^	T	2.73	1.24, 6.03	0.013	0.091
**Normal-like tumor subtype**						
Non-normal-like			Reference	Reference	Reference	Reference
Normal-like	rs5940	T	22.17	4.43, 110.8	0.0002	0.003

^a^SNPs representing a haplotype effect. SNPs are listed by ascending chromosome positions. TFPI, tissue factor pathway inhibitor; ER, estrogen receptor; PR, progesterone receptor; HER2, human epidermal growth factor 2.

The G-C-T-C haplotype (frequency 0.42) formed by the four SNPs (rs3213739-rs8176479-rs2192824-rs10187622) was associated with the basal tumor subtype with OR 2.3 (*P* = 0.011) (Table 3). The three-SNP haplotype T-G-T (rs16829086-rs10179730-rs10187622) was associated with the luminal B subtype (OR 3.5, *P* = 0.005, frequency 0.09), and rs5940 was associated with the normal-like subtype (OR 22.2, *P* = 0.0002). No associations between *TFPI* SNPs and tumor grade, PR-status, HER2 status, the HER2-enriched subtype or the luminal A subtype were observed. *TF* SNPs did not associate with any of the clinicopathological characteristics or molecular tumor subtypes herein evaluated (data not shown). Age did not correlate to any of the SNP genotypes and adjusting for age did not affect the associations (data not shown).

### Associations between SNPs and TFPI and TF expression

Further, we aimed to explore whether the *TFPI* or the *TF* SNPs had any transcriptional or translational regulatory effects on the *TFPI* or *TF* mRNA expression in the breast tumors, or on the protein levels in plasma, respectively. A total of six intronic *TFPI* SNPs correlated to TFPIβ mRNA expression (Table 4), and accompanied by conditional association analysis, the LD pattern between the six SNPs encouraged haplotype analysis. The T-A-C-C-T-C haplotype (frequency 0.03), made up of the minor alleles of the six SNPs (rs3213739-rs8176479-rs2192824-rs12613071-rs2192825-rs7594359 correlated positively to TFPIβ mRNA (B = 0.76, *r* = 0.23, *P* = 0.004). Among these six SNPs, three also correlated to total TFPI (α + β) mRNA expression (rs2192824-rs12613071-rs7594359) with an inverse correlation of *r* = −0.22 (*B* = −0.25, *P* = 0.008) for the T-T-T haplotype (frequency 0.40). Furthermore, the minor allele T-T haplotype formed by two of these SNPs (rs2192824-rs7594359) correlated inversely to TFPIα mRNA expression with *r* = −0.23 (*B* = −0.29, *P =* 0.004). No significant correlation between *TF* SNPs and TF mRNA expression was observed (*P* >0.05, data not shown).

**Table 4:** **Significant correlations between** ***TFPI*** **single nucleotide polymorphisms (SNPs) and** ***TFPI*** **mRNA expression in breast tumors**

**Probe**	**SNP**	**Region**	**Alleles** ^**a**^	**Minor allele frequency**	**Beta**	**r**	***P***	**False discovery rate**
TFPIα	rs2192824^b^	Intronic	C:T	0.490	−0.209	−0.180	0.029	0.200
TFPIα	rs7594359^b^	Intronic	C:T	0.483	−0.219	−0.184	0.025	0.200
TFPIβ	rs3213739^b^	Intronic	G:T	0.417	0.187	0.213	0.010	0.032
TFPIβ	rs8176479^b^	Intronic	C:A	0.238	0.184	0.192	0.021	0.049
TFPIβ	rs2192824^b^	Intronic	C:T	0.490	−0.267	−0.273	0.001	0.011
TFPIβ	rs12613071^b^	Intronic	T:C	0.158	0.284	0.208	0.011	0.032
TFPIβ	rs2192825^b^	Intronic	T:C	0.466	−0.251	−0.249	0.002	0.012
TFPIβ	rs7594359^b^	Intronic	C:T	0.483	−0.248	−0.247	0.002	0.012
TFPIα + β	rs2192824^b^	Intronic	C:T	0.490	−0.168	−0.161	0.050	0.187
TFPIα + β	rs12613071^b^	Intronic	T:C	0.158	0.238	0.164	0.048	0.187
TFPIα + β	rs7594359^b^	Intronic	C:T	0.483	−0.190	−0.178	0.030	0.187

^a^Major:minor. ^b^SNPs representing a haplotype effect**.** mRNA expression was assayed by the Agilent Human V2 Gene Expression 8x60k array, and probes for tissue factor pathway inhibitor (TFPI)α, TFPIβ and total TFPI (TFPIα + β) mRNA were analyzed. Alleles for the positive DNA strand (UCSC annotated) are shown, and SNPs are listed by ascending chromosome positions.

Eight *TFPI* SNPs were found to be correlated to total TFPI protein levels in patient plasma (Table 5). The A-T-A-C-T-A-C-G haplotype composed of these eight SNPs (rs8176541-rs3213739-rs8176479-rs2192824-rs2192825-rs16829088-rs7594359-rs10153820) represented a common haplotype (frequency 0.19) with quite strong correlation to total TFPI protein; *r* = 0.481 (*B* = 14.62, *P* = 6.35 × 10^−10^). No correlation between *TFPI* SNPs and free TFPI protein, or between *TF* SNPs and TF protein in plasma was observed (*P* >0.05, data not shown). Adjusting for age had no effect on the correlation (data not shown).

**Table 5:** **Significant correlations between** ***TFPI*** **single nucleotide polymorphisms (SNPs) and total TFPI protein levels in plasma**

**Protein**	**SNP**	**Region**	**Alleles** ^**a**^	**Minor allele frequency**	**Beta**	***r***	***P***	**False discovery rate**
Total TFPI	rs8176541^b^	Intronic	G:A	0.283	15.64	0.571	7.69 × 10^−14^	1.08 × 10^−12^
Total TFPI	rs3213739^b^	Intronic	G:T	0.417	11.35	0.488	5.38 × 10^−10^	3.77 × 10^−9^
Total TFPI	rs8176479^b^	Intronic	C:A	0.238	12.22	0.480	1.20 × 10^−9^	5.62 × 10^−9^
Total TFPI	rs2192824^b^	Intronic	C:T	0.490	−9.88	−0.404	3.81 × 10^−7^	1.07 × 10^6^
Total TFPI	rs2192825^b^	Intronic	T:C	0.466	−7.55	−0.301	2.40 × 10^−4^	5.30 × 10^−4^
Total TFPI	rs16829088^b^	Intronic	G:A	0.250	11.23	0.424	1.00 × 10^−7^	3.51 × 10^−7^
Total TFPI	rs7594359^b^	Intronic	C:T	0.483	−6.90	−0.275	6.90 × 10^−4^	0.001
Total TFPI	rs10153820^b^	Near 5UTR	G:A	0.125	−7.79	−0.215	0.009	0.016

^a^Major:minor. ^b^SNPs representing a haplotype effect for total tissue factor pathway inhibitor (TFPI). Alleles for the positive DNA strand (UCSC annotated) are shown.

### TFPI and TF tumor mRNA expression and survival

**Figure 3:**
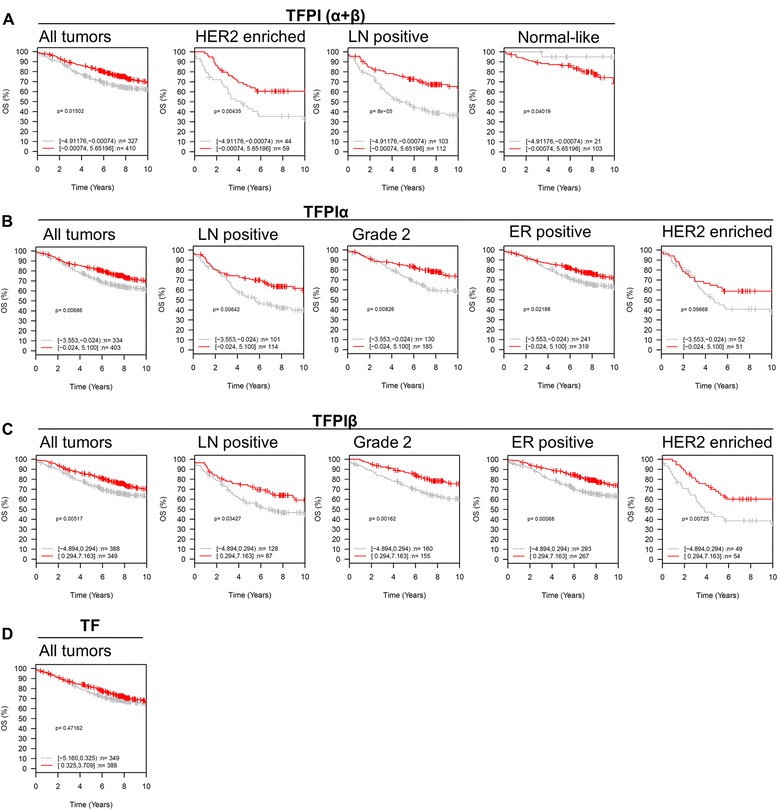
**Overall survival in breast cancer patients stratified by tissue factor pathway inhibitor (TFPI) and tissue factor (TF) expression in tumors.** Kaplan-Meier survival curve with overall survival (OS) with 10-year censoring as endpoint, stratified according to high (above the median) and low (below median) total *TFPI* (α + β) gene expression levels **(A)**, *TFPI*α^a^ gene expression levels **(B)**, *TFPI*β gene expression levels **(C)**, and *TF* gene expression levels **(D)**, in all tumors and selected clinical subgroups in which survival data were available. Analyses were performed using the Gene expression based Outcome for Breast cancer Online (GOBO) database, and the log-rank test was used to calculate *P*-values. ^a^For *TFPI*α expression results were obtained by merging the two available probe sets, as specified in Additional file 4: Table S3.

**Figure 4:**
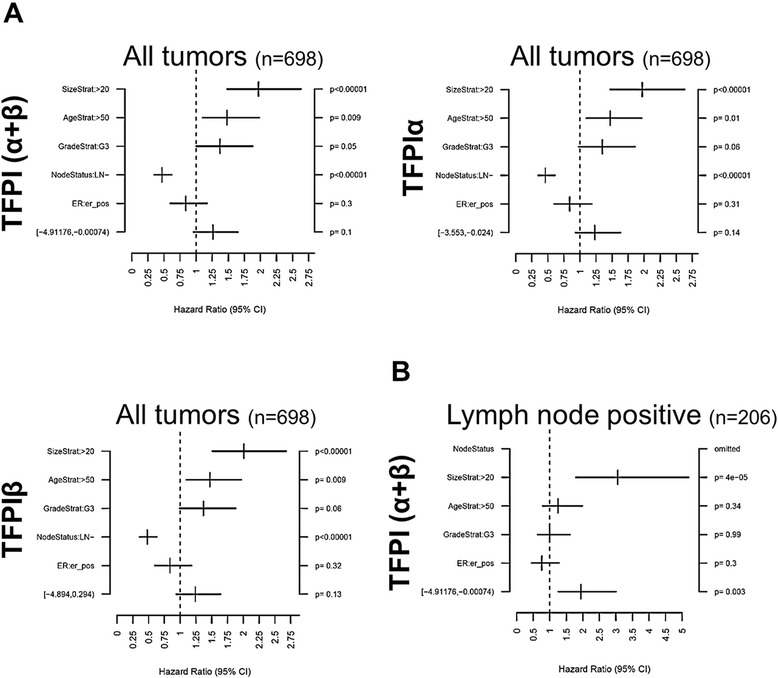
**Multivariate survival analysis in breast cancer patients stratified by tissue factor pathway inhibitor (TFPI) and tissue factor (TF) expression in tumors.** Multivariate survival analysis derived from the Gene expression based Outcome for Breast cancer Online (GOBO) database of **(A)** total TFPI (α + β), TFPIα, and TFPIβ tumor expression in all tumors, and **(B)** total TFPI (α + β) tumor expression in the lymph node-positive group. Tumor size, age, histological tumor grade, lymph node status^a^ and estrogen receptor (ER) status were included as covariates. Hazard ratios, 95% CIs and corresponding *P*-values are specified for each covariate. ^a^Omitted from the analysis of the lymph node-positive group.

Survival data were not available for our patient cohort, however, access to a merged breast cancer dataset from GOBO allowed us to investigate possible associations between *TFPI* or *TF* gene expression and outcome. Low expression of total TFPI (α + β) showed significant association with decreased overall survival when considering all breast cancer subtypes together (all tumors) (*P* = 0.015), and the effect was even more profound in the high-proliferative poor outcome classes; HER2-enriched (*P* = 0.004) and lymph node-positive cancers (*P* = 8*10^−5^) (Figure 3A). A survival effect was also observed for normal-like tumors (*P* = 0.040). Similarly, low expression of TFPIα and TFPIβ were predictive of reduced overall survival in all tumors (*P* = 0.007 and 0.005), lymph node-positive (*P* = 0.006 and 0.034), grade-2 (*P* = 0.008 and 0.001), ER-positive (*P* = 0.022 and 0.0007), and HER2-enriched tumors (*P* = 0.097 (not significant) and 0.007) (Figure 3B and C). Similar patterns were observed for relapse-free survival (Additional file 8: Figure S5). Furthermore, multivariate hazard ratio analysis of all tumors, showed that tumor size and lymph node status were highly associated with overall survival (*P* <0.00001) and abolished the effect of low TFPI expression (*P* >0.05) (Figure 4A). This result implied that the survival effect of TFPI expression involves lymph node metastasis and proliferation (reflected by tumor size), thus providing a possible explanation for why low TFPI expression had an even greater impact to the subgroup-stratified survival analysis, compared to analysis of all tumors (Figure 3A-C). This was further demonstrated by multivariate analyses in the lymph node-positive group. This covariate-adjusted analysis showed that low total TFPI (α + β) expression was a prognostic indicator for overall survival, independent of the strong association with tumor size (Figure 4B). Notably, TF expression rates did not correlate with overall survival in all tumors (Figure 3D) or in any of the clinical subgroups, nor with relapse-free survival (data not shown).

## Discussion

TF and TFPI exert opposing effects in coagulation, but also in cancer biology; although TF seems to be a tumor-promoting factor, TFPI exerts tumor suppressor activities. The mechanisms in which these actions take place, however, are not fully elucidated.

Here, we report several indications that TFPI have clinical significance in breast cancer. First, mRNA expression of both total TFPI (α + β) and TFPIβ were significantly associated with PR negativity. Our study lacked power to detect an association with ER negativity due to a smaller number of ER-negative (n = 35) than PR-negative (n = 51) patients. However, PR-negativity is a surrogate marker of a non-functioning ER pathway [41]. There was also a tendency towards increased mRNA expression of either total TFPI (α + β) or TFPIα or TFPIβ in highly proliferative and poor-outcome groups, such as those with larger tumors and HER2 positivity, in keeping with the survival analysis discussed later. In accordance with the IHC determination of HER2 status, enhanced TFPI expression was evident also in the HER2-enriched molecular subgroup (in the merged dataset). Moreover, TFPI expression tended to be increased in tumors of patients with lymph node metastases, supporting the results and the proposal of Sierko *et al.* that TFPI localized in regional lymph nodes may indicate a role for TFPI in lymphatic spread [9]. Additionally, tumor-expressed TFPI and TF were positively correlated, which is in line with our previous findings in breast cancer cell lines that TFPI expression follows TF expression and the aggressive basal-like tumor subtype [7]. Stavik *et al.* proposed a role for TFPI in cancer progression as they showed that overexpression of TFPIα or TFPIβ in breast cancer cell lines affected expression of genes involved in cancer-related cellular functions (for example, proliferation, migration, and invasion). Additionally, they revealed that the gene signatures following overexpression of either TFPI isoform were associated with tumor grade and ER status, and that TFPIα expression correlated with tumor size [42].

Taken together, we hypothesize that TFPI is a marker of more aggressive tumors while at the same time being a tumor suppressor. Paradoxical as it may seem, these two hypotheses are not mutually exclusive. This is because TFPI has previously been recognized as a tumor suppressor in experimental studies [16-21], and because higher TFPI expression in tumors was associated with increased survival in the present study. TFPI expression in tumors may then serve to oppose and protect against tumor progression. TFPI may therefore represent a prognostic marker, but also an anti-cancer candidate with potential for being translated into the clinic.

Contrasting the observations we made in tumors, the plasma levels of total TFPI tended to be lower in larger tumors, triple-negative tumors, and grade-3 tumors. These results comply with the lack of correlation between TFPI tumor expression and TFPI plasma levels, suggesting that the systemic plasma concentrations of TFPI were not influenced by or derived from the tumor cells and thereby cannot predict plasma levels of TFPI. Yet, no directly comparable data are available, but Lindahl *et al.* reported that TFPI activity in plasma increased in parallel with gastrointestinal cancer progression [43]. Although we measured levels of TFPI instead of activity, this suggests that the behavior of plasma TFPI proceeds differently in different cancers. Later, Iversen *et al.* reported that the median TFPI activity levels were within the normal range in breast cancer. These authors also observed that TFPI activity was higher in metastatic cancer patients [44]. In our study none of the patients had developed distant metastases at the time of inclusion.

The complexity of the different forms of circulating TFPI (that is, full-length TFPI, C-terminal truncated, and lipid-bound) [15] was illustrated, as free TFPI, unlike total TFPI, could not be designated as having any clinical relevance. In line with this, no differences in free TFPI plasma levels were detected between breast cancer patients and controls in our preceding study [45].

We identified several *TFPI* SNPs (with haplotype effects) that were more frequent in specific clinicopathological tumor characteristics, such as tumor size, triple-negative status and lymph node status, as well as basal and luminal B tumor subtypes. Although the precise role for these SNP associations is still to be elucidated, this study pinpoints the importance of focusing on breast cancer heterogeneity, as in a recent study we found that the frequencies of *TFPI* SNPs in a group of breast cancer patients with all subtypes combined (n = 366), were no different from healthy control subjects [45]. Some of the SNPs that followed distinct clinical groups (rs3213739, rs8176479, rs2192824, rs8176541 and rs10153820) also correlated with total TFPI (α + β), TFPIα or TFPIβ tumor mRNA expression, or total TFPI plasma levels. Certain SNPs with a regulatory effect on total TFPI plasma levels also associated with triple-negative status (rs8176541, rs3213739, rs8176479 and rs2192824) and tumor size (rs10153820), thereby providing a possible mechanistic rationale for why total TFPI levels were decreased in patients with triple-negative and larger tumors. However, the exact mechanism(s) by which these SNPs regulate TFPI protein levels demand experimental verification. Most SNPs in this study were positioned in introns, but many of them were in strong LD with SNPs located in 3′ UTR and 5′ UTR (not shown), which are regions known to have potential influence on gene expression.

The minor allele of rs10153820 (−399C/T) was associated with decreased total TFPI plasma levels, and the minor allele of rs8176541, a perfect predictor of rs8176592 (−33 T/C) (pairwise LD *r*^2^ = 1.00), correlated to higher total TFPI plasma levels. This result is in line with another study that genotyped rs8176592 [46], whilst another study failed to show an effect of this SNP on total TFPI plasma levels [29].

In light of the abundant TF expression in cancers [6-8,10] and involvement in angiogenesis, metastasis and tumor growth [11-14], the virtual absence of clinical significance of TF tumor mRNA expression or plasma levels, or *TF* genetic polymorphisms was not anticipated, that is, with the exception of a reverse association between TF mRNA tumor expression and tumor grade. This result is in conflict with Kocatürk *et al.* who found positive correlation between TF and grade [6], but in agreement with the merged GOBO breast cancer dataset in this study. We could hypothesize that tumor expressed TF may be more important in the earlier phases of malignant transformation, as suggested in pancreatic cancer [47].

In contrast to the experimental evidence that points to TF as an important contributor in tumor progression, studies investigating the clinical relevance of tissue factor in breast cancer patients have generated less conclusive results. Adding to the hypothesis that a procoagulant state extends beyond the risk of venous thromobosis (VT), Hernández *et al.* reported that increased TF plasma levels and activity in a number of cancers, amongst them breast cancer, was indicative of worse prognosis, but not of VT [48]. Also Ueno *et al.* demonstrated that TF plasma levels were elevated in breast cancer patients compared to controls. However, neither TF plasma levels nor tumor expression were found related to clinical stage, HR status or lymph-node metastasis [8]. Yet another study failed to detect any correlation between breast tumor-expressed TF and HR status, node status, grade or tumor size [10]. Kocatürk *et al*., found that both flTF and alternatively spliced TF (asTF) [GenBank:NM_001178096] were associated with increasing tumor grade, while asTF was also associated with increasing breast tumor size [6], suggesting that the clinical significance of asTF is at least as important as that of flTF. Consequently, future studies addressing TF in relation to cancer should include the different forms of TF (that is, asTF and flTF [6,49], cryptic versus active TF [50], and phosphorylated TF [10]). Also noteworthy; methodological discrepancies in measuring TF may to some extent account for the conflicting results among studies addressing TF in cancer (for example, mRNA versus protein and use of different TF detection antibodies).

In clinical cancer research, death is a common endpoint of interest. Because survival data were not available for the patients included in our study, we made use of a merged clinical dataset to investigate the effect of TFPI and TF tumor expression on survival among breast cancer patients. No difference in overall or relapse-free survival was observed between patients with high or low levels of TF. This is somehow contradictory to the study of Ueno *et al.* who found that breast cancer patients with TF-positive tumors were associated with reduced overall survival, however, not with disease-free survival [8]. On the contrary, no previous studies have described survival data directly in relation to tumor expressed TFPI, but Stavik *et al.* reported that the gene expression signature resulting from TFPIβ overexpression correlated with relapse-free survival in breast cancer [42]. We now report that breast cancer patients with low tumor expression of both isoforms of TFPI had worse outcome in terms of both overall- and relapse free survival compared to patients with higher TFPI expression. Therefore, it seems that increased tumor expression of TFPI may be beneficial in breast cancer.

## Conclusions

Here we provide results suggesting that TFPI represents a promising marker of breast cancer progression and prognosis. This was especially evident in clinically relevant groups such as lymph node-positive patients. The clinical relevance of expression and genetic variants of *TFPI* in breast cancer appeared distinct from that of TF. Together with the previously demonstrated tumor suppressor effects of TFPI, the beneficial survival effect of tumor-expressed TFPI highlights the potential of TFPI as a candidate in cancer therapy, and this clearly deserves further investigation for possible translation to clinical practice.

## Additional files

Additional file 1: Table S1. Probes from the Agilent Human V2 Gene Expression 8x60k array in which results were used in the study. SNPs occurring in probes are shown in bold. Additional file 2: Table S2. All analyzed single nucleotide polymorphisms (SNPs) in tissue factor pathway inhibitor (*TFPI)* and tissue factor (*TF)* genes. Alleles for the positive strand are shown (UCSC-annotated). Additional file 3: Figure S1. Linkage disequilibrium (LD) map of all fourteen *TFPI* SNPs included in the study. Dʹ values are shown. Additional file 4: Table S3. Probe set regions for the Affymetrix U133A microarray used in the study. Additional file 5: Figure S2. Expression heatmaps for A) total TFPI (α + β), TFPIβ and TF mRNA expression in breast tumors differentiated by clinically relevant subgroups of PR-status (PR negative and PR positive) and tumor grade (G1 + 2 and G3), and B) plasma levels of total TFPI according to T-status (T1 and T2 + T3). The color key is indicated. Additional file 6: Figure S3. Box and Whiskers plot showing the distribution of log2 transformed total TFPI (α + β), TFPIα, and TFPIβ and *TF* tumor mRNA expression across the following PAM50 subtype signatures of 150* breast cancer patients; basal (n = 304/357), HER2 enriched (n = 240/152), luminal A (n = 465/482), luminal B (n = 471/289) and normal-like (n = 304/257). Data were derived from the GOBO database. *P*-values for ANOVA testing are indicated. Additional file 7: Figure S4. Box and Whiskers plot showing the distribution of *TF* gene expression for tumor samples across the three classes of histological grade. Expression rates are analyzed by Affymetrix Human Genome U133A arrays and data were derived from all tumors of the GOBO database. Additional file 8: Figure S5. Kaplan-Meier survival curve with relapse-free survival (RFS) with 10-year censoring as the endpoint, stratified according to high (above the median) and low (below median) total tissue factor pathway inhibitor (*TFPI*) (α + β) gene expression levels **(A)**, *TFPIα* ^a^ gene expression levels **(B)**, and *TFPIβ* gene expression levels **(C)**, in all tumors and selected clinical subgroups in which survival data were available. Analyses were performed using the Gene expression based Outcome for Breast cancer Online (GOBO) database, and the log-rank test was used to calculate *P*-values. ^a^For TFPIα expression results were obtained by merging the two available probe sets, as specified in Additional file 4: Table S3.

## Abbrevations

asTF: alternatively spliced tissue factor
ANOVA: analysis of variance
ELISA: enzyme-linked immunosorbent assay
FDR: false discovery rate
GOBO: Gene expression based Outcome for Breast cancer Online
ER: estrogen receptor
HE: hematoxylin and eosin
HER2: human epidermal growth factor receptor 2
HR: hormone receptor
IHC: immunohistochemistry
LD: linkage disequilibrium
MAF: minor allele frequency
OR: odds ratio
PR: progesterone receptor
PAM50: prediction analysis of microarray 50
SNP: single nucleotide polymorphism
TF: tissue factor
TFPI: tissue factor pathway inhibitor
TN: triple negative
UTR: untranslated region
VT: venous thromobosis
