# Supplementary material for: Tumor expression, plasma levels and genetic polymorphisms of the coagulation inhibitor TFPI are associated with clinicopathological parameters and survival in breast cancer, in contrast to the coagulation initiator TF
Source: Breast Cancer Res. 2015 Mar 26;17(1):44. doi: 10.1186/s13058-015-0548-5 (PMC4423106; doi:10.1186/s13058-015-0548-5)
Supplement: Additional file 1: Table S1. — Probes from the Agilent Human V2 Gene Expression 8x60k array in which results were used in the study. SNPs occurring in probes are shown in bold. [file 13058_2015_548_MOESM1_ESM.pdf]

Supplementary Table S1. Probes from the Agilent Human V2 Gene Expression 8x60k array in which results were used in the study. SNPs occurring in probes are shown in **bold.**

**Probe**	**Probe sequence (5′→3′)**	**Gene (isoform)**	**Reference no.**	**Position (hg19)**
A_33_P3212615	CTGCTTTGTTCATTCTCTGTA<br>TTCAAAATTAACAGA**T**CATG<br>CTGAAAACCACTCAAACGA	*TFPI* (α)	NM_006287	chr2:<br>188329067-<br>188329008
A_23_P330070	GCCTTCTGCATTCATGCATCC<br>ATGTTCTTTCTAGGATTGGAT<br>AGCATTTCATGCCTATGT	*TFPI* (β)	NM_001032281	chr2:<br>188343465-<br>188343406
A_33_P3258274	GAGACACTGGAAGAATGCAA<br>GAACATTTGTGAAGATGGTC<br>GTAAGTTTATTTCT**T**ATTTC	*TFPI* (total)	ENST00000374896[a]	chr2:<br>188349577-<br>188349518
A_33_P3226832	CCTAATATGCTTTACAATCTG<br>CACTTTAACTGACTTAAGTGG<br>CATTA**A**AC**A**TTTGAGAGC	*TF*[b]<br>(fl*TF* and<br>as*TF*)	NM_001993 (flTF)<br>NM_001178096 (asTF)	chr1:<br>94995139-<br>94995080

[a]Retired from the current ENSEMBL genome browser
[b]*F3*
