# Supplementary material for: Tumor expression, plasma levels and genetic polymorphisms of the coagulation inhibitor TFPI are associated with clinicopathological parameters and survival in breast cancer, in contrast to the coagulation initiator TF
Source: Breast Cancer Res. 2015 Mar 26;17(1):44. doi: 10.1186/s13058-015-0548-5 (PMC4423106; doi:10.1186/s13058-015-0548-5)
Supplement: Additional file 2: Table S2. — All analyzed single nucleotide polymorphisms (SNPs) in tissue factor pathway inhibitor (TFPI) and tissue factor (TF) genes. Alleles for the positive strand are shown (UCSC-annotated). [file 13058_2015_548_MOESM2_ESM.pdf]

Supplementary Table S2. All analyzed SNPs in *TFPI* and *TF*. Alleles for the positive strand are shown (UCSC annotated).

SNP	Gene	Position (hg19)	Region	Alleles[a]	MAF[b]
rs5940	*TFPI*	chr2:188331704	Coding (V>M)	C:T	0.034
rs8176541[t]	*TFPI*	chr2:188341047	Intron	G:A	0.283
rs3213739[t]	*TFPI*	chr2:188348670	Intron	G:T	0.417
rs8176605[t]	*TFPI*	chr2:188356110	Intron	A:G	0.062
rs8176479[t]	*TFPI*	chr2:188357179	Intron	C:A	0.238
rs2192824	*TFPI*	chr2:188368791	Intronic	C:T	0.490
rs12613071	*TFPI*	chr2:188388311	Intronic	T:C	0.158
rs2192825	*TFPI*	chr2:188390819	Intronic	T:C	0.466
rs16829086	*TFPI*	chr2:188391250	Intronic	C:T	0.192
rs16829088	*TFPI*	chr2:188391908	Intronic	G:A	0.250
rs7594359	*TFPI*	chr2:188408848	Intronic	C:T	0.483
rs10179730	*TFPI*	chr2:188410351	Intronic	A:G	0.091
rs10187622	*TFPI*	chr2:188414161	Intronic	C:T	0.134
rs10153820	*TFPI*	chr2:188419590	Near 5UTR	G:A	0.125
rs3917643	*TF*	chr1:95001867	Intronic	T:C	0.051
rs762485	*TF*	chr1:95004084	Intronic	A:C	0.447
rs762484	*TF*	chr1:95004410	Intronic	T:C	0.238
rs696619	*TF*	chr1:95005220	Intron	G:A	0.413
rs1361600	*TF*	chr1:95007918	Near 5UTR	T:C	0.480
rs958587	*TF*	chr1:95009145	Near 5UTR	G:A	0.480

[a]Major:minor
[b]MAF= minor allele frequency
[t]SNPs genotyped in tumor-derived tissue.
