# Supplementary material for: Tumor expression, plasma levels and genetic polymorphisms of the coagulation inhibitor TFPI are associated with clinicopathological parameters and survival in breast cancer, in contrast to the coagulation initiator TF
Source: Breast Cancer Res. 2015 Mar 26;17(1):44. doi: 10.1186/s13058-015-0548-5 (PMC4423106; doi:10.1186/s13058-015-0548-5)

Supplementary Figure S1

Linkage disequilibrium (LD) map of all fourteen *TFPI* SNPs included in the study. D′ values are shown.

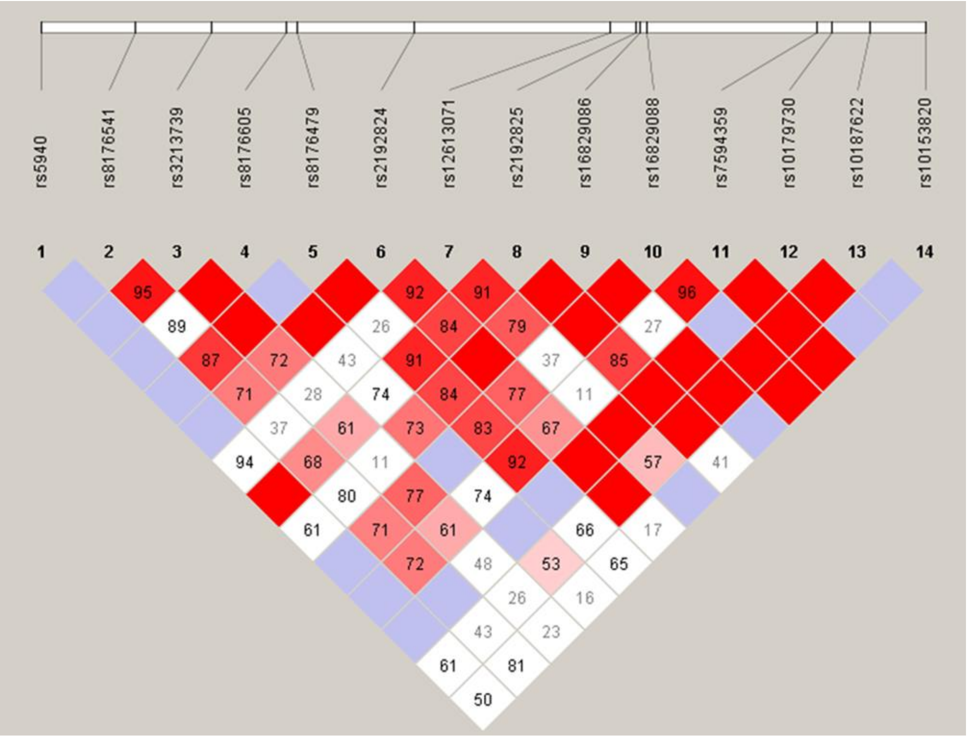

Supplement: Additional file 3: Figure S1. — Linkage disequilibrium (LD) map of all fourteen TFPI SNPs included in the study. Dʹ values are shown. [file 13058_2015_548_MOESM3_ESM.pdf]
