# Supplementary material for: Tumor expression, plasma levels and genetic polymorphisms of the coagulation inhibitor TFPI are associated with clinicopathological parameters and survival in breast cancer, in contrast to the coagulation initiator TF
Source: Breast Cancer Res. 2015 Mar 26;17(1):44. doi: 10.1186/s13058-015-0548-5 (PMC4423106; doi:10.1186/s13058-015-0548-5)
Supplement: Additional file 4: Table S3. — Probe set regions for the Affymetrix U133A microarray used in the study. [file 13058_2015_548_MOESM4_ESM.pdf]

Supplementary Table S3. Probe set regions for the Affymetrix U133A microarray used in the study.

Probe set	Target sequence (hg19)	Gene isoform	Reference no.
213258_at	chr2: 188329009-188329543	*TFPI* (α)	NM_006287
209676_at	chr2:188331349-188332581	*TFPI* (α)	NM_006287
210665_at	chr2: 188343336 188343514	*TFPI* (β)	NM_001032281
210664_s_at	chr2: 188348864-188349710	*TFPI* (α+β)	NM_006287 and NM_001032281
204363_at	chr1:94994997-94995395	*TF*[a] (fl*TF* + as*TF*)	NM_001993 (flTF) and NM_001178096 (asTF)

[a]*F3*
