# Supplementary material for: Tumor expression, plasma levels and genetic polymorphisms of the coagulation inhibitor TFPI are associated with clinicopathological parameters and survival in breast cancer, in contrast to the coagulation initiator TF
Source: Breast Cancer Res. 2015 Mar 26;17(1):44. doi: 10.1186/s13058-015-0548-5 (PMC4423106; doi:10.1186/s13058-015-0548-5)

Supplementary Figure S2

Expression heatmaps for A) total TFPI (α+β), TFPIβ and TF mRNA expression in breast tumors differentiated by clinically relevant subgroups of PR-status (PR negative and PR positive) and tumor grade (G1+2 and G3), and B) plasma levels of total TFPI according to T-status (T1 and T2+T3). The color key is indicated.

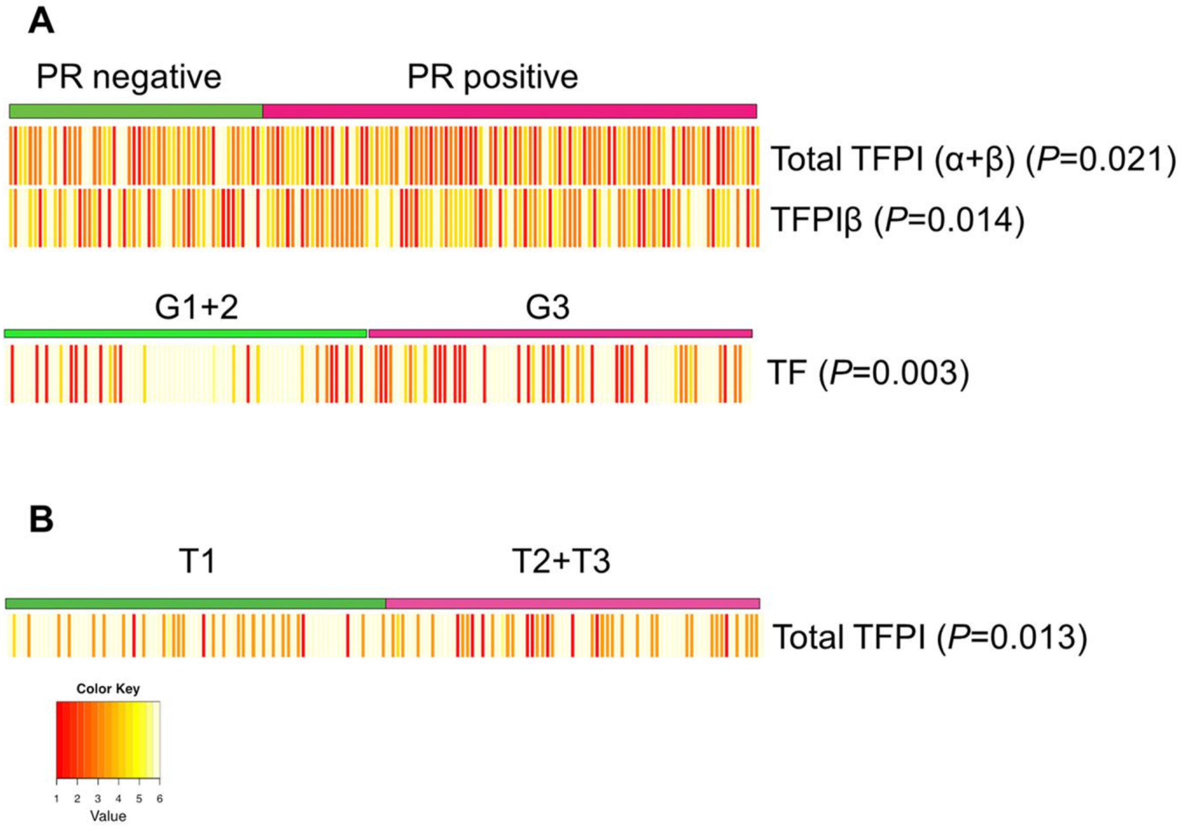

Supplement: Additional file 5: Figure S2. — Expression heatmaps for A) total TFPI (α + β), TFPIβ and TF mRNA expression in breast tumors differentiated by clinically relevant subgroups of PR-status (PR negative and PR positive) and tumor grade (G1 + 2 and G3), and B) plasma levels of total TFPI according to T-status (T1 and T2 + T3). The color key is indicated. [file 13058_2015_548_MOESM5_ESM.pdf]
