# Supplementary material for: Tumor expression, plasma levels and genetic polymorphisms of the coagulation inhibitor TFPI are associated with clinicopathological parameters and survival in breast cancer, in contrast to the coagulation initiator TF
Source: Breast Cancer Res. 2015 Mar 26;17(1):44. doi: 10.1186/s13058-015-0548-5 (PMC4423106; doi:10.1186/s13058-015-0548-5)

Supplementary Figure S3

Box and Whiskers plot showing the distribution of log2 transformed total TFPI (α+β), TFPIα, and TFPIβ and *TF* tumor mRNA expression across the following PAM50 subtype signatures of 150* breast cancer patients; basal (n=304/357), HER2 enriched (n=240/152), luminal A (n=465/482), luminal B (n=471/289) and normal-like (n=304/257). Data were derived from the GOBO database. *P*-values for ANOVA testing are indicated.

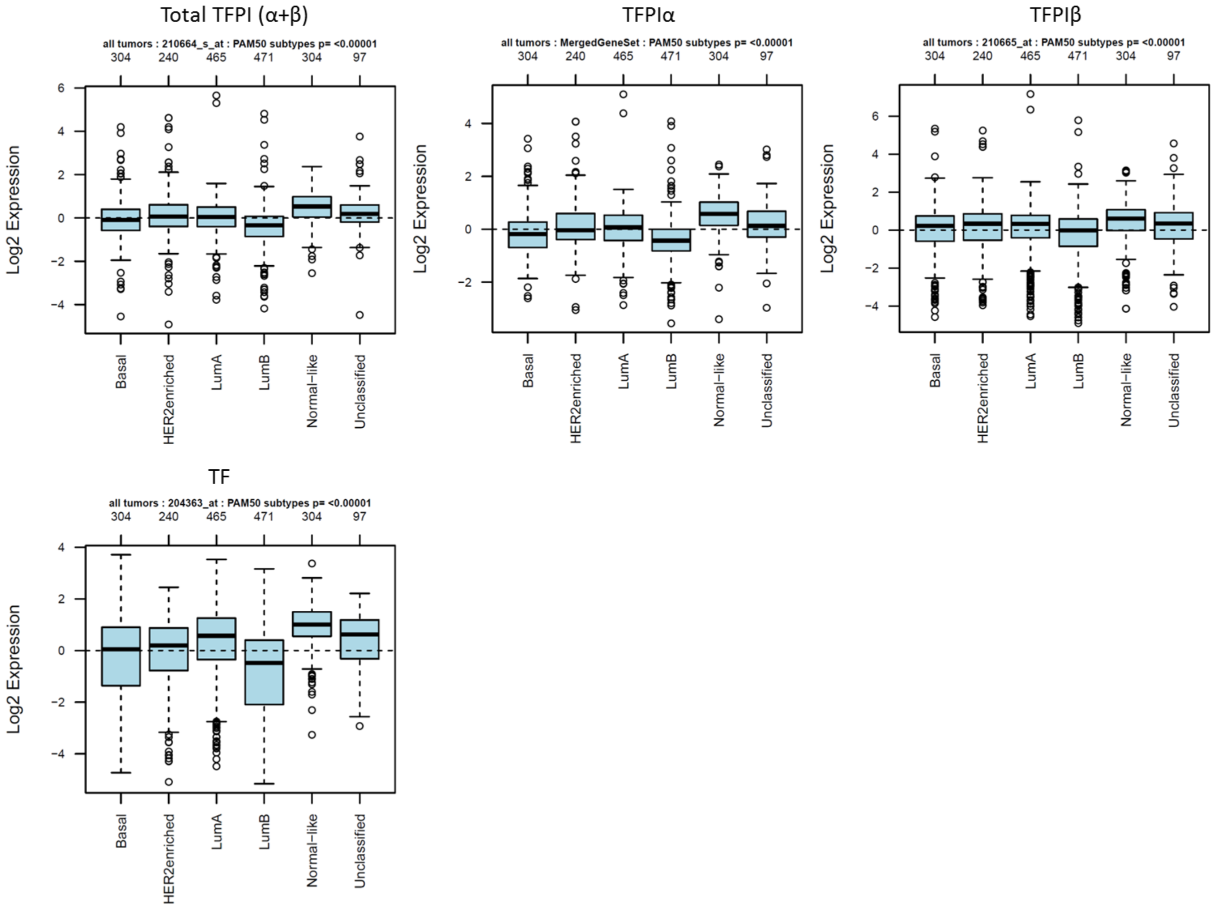

Supplement: Additional file 6: Figure S3. — Box and Whiskers plot showing the distribution of log2 transformed total TFPI (α + β), TFPIα, and TFPIβ and TF tumor mRNA expression across the following PAM50 subtype signatures of 150* breast cancer patients; basal (n = 304/357), HER2 enriched (n = 240/152), luminal A (n = 465/482), luminal B (n = 471/289) and normal-like (n = 304/257). Data were derived from the GOBO database. P-values for ANOVA testing are indicated. [file 13058_2015_548_MOESM6_ESM.pdf]
