# Supplementary material for: Tumor expression, plasma levels and genetic polymorphisms of the coagulation inhibitor TFPI are associated with clinicopathological parameters and survival in breast cancer, in contrast to the coagulation initiator TF
Source: Breast Cancer Res. 2015 Mar 26;17(1):44. doi: 10.1186/s13058-015-0548-5 (PMC4423106; doi:10.1186/s13058-015-0548-5)

Supplementary Figure S4

Box and Whiskers plot showing the distribution of *TF* gene expression for tumor samples across the three classes of histological grade. Expression rates are analyzed by Affymetrix Human Genome U133A arrays and data were derived from all tumors of the GOBO database.

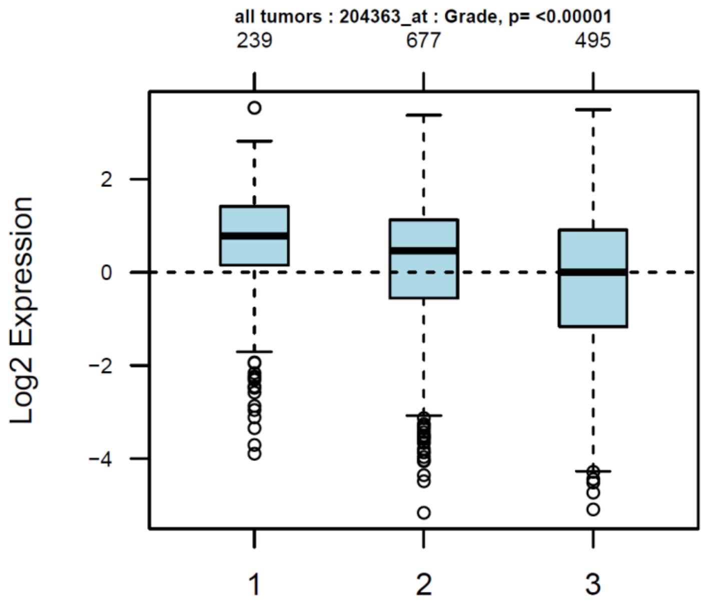

Supplement: Additional file 7: Figure S4. — Box and Whiskers plot showing the distribution of TF gene expression for tumor samples across the three classes of histological grade. Expression rates are analyzed by Affymetrix Human Genome U133A arrays and data were derived from all tumors of the GOBO database. [file 13058_2015_548_MOESM7_ESM.pdf]
