# Supplementary material for: Tumor expression, plasma levels and genetic polymorphisms of the coagulation inhibitor TFPI are associated with clinicopathological parameters and survival in breast cancer, in contrast to the coagulation initiator TF
Source: Breast Cancer Res. 2015 Mar 26;17(1):44. doi: 10.1186/s13058-015-0548-5 (PMC4423106; doi:10.1186/s13058-015-0548-5)
Supplement: Additional file 8: Figure S5. — Kaplan-Meier survival curve with relapse-free survival (RFS) with 10-year censoring as the endpoint, stratified according to high (above the median) and low (below median) total tissue factor pathway inhibitor (TFPI) (α + β) gene expression levels (A), TFPIα a gene expression levels (B), and TFPIβ gene expression levels (C), in all tumors and selected clinical subgroups in which survival data were available. Analyses were performed using the Gene expression based Outcome for Breast cancer Online (GOBO) database, and the log-rank test was used to calculate P-values. aFor TFPIα expression results were obtained by merging the two available probe sets, as specified in Additional file 4: Table S3. [file 13058_2015_548_MOESM8_ESM.pdf]

Supplementary Figure S5

Kaplan-Meier survival curve with relapse free survival (RFS) with 10-year censoring as endpoint, stratified according to high (above the median) and low (below median) total TFPI (α+β) gene expression levels (A), TFPIα[a] gene expression levels (B), and TFPIβ gene expression levels (C), in all tumors and selected clinical subgroups in which survival data was available. Analyzes were performed using the GOBO database, and the log-rank test was used to calculate *P*-values.

[a]For TFPIα expression results were obtained by merging the two available probe sets, as specified in Additional file 4: Table S3.

A

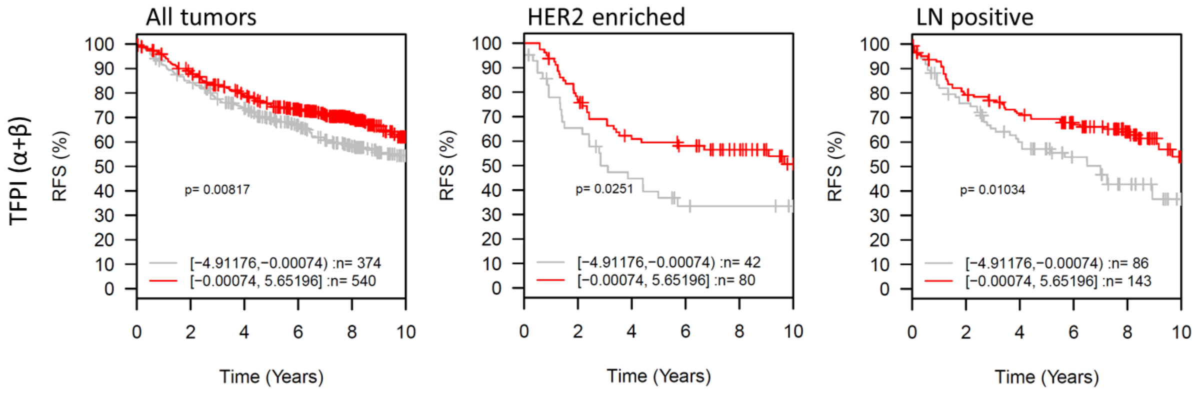

B

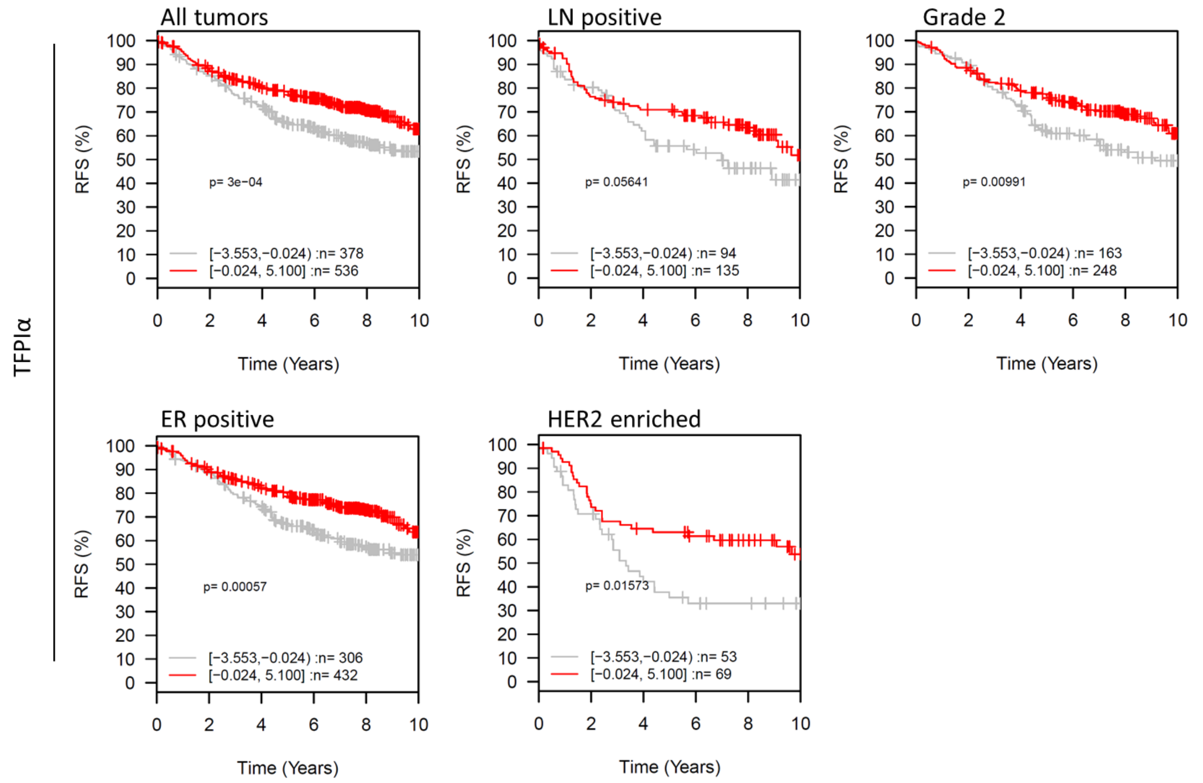

C

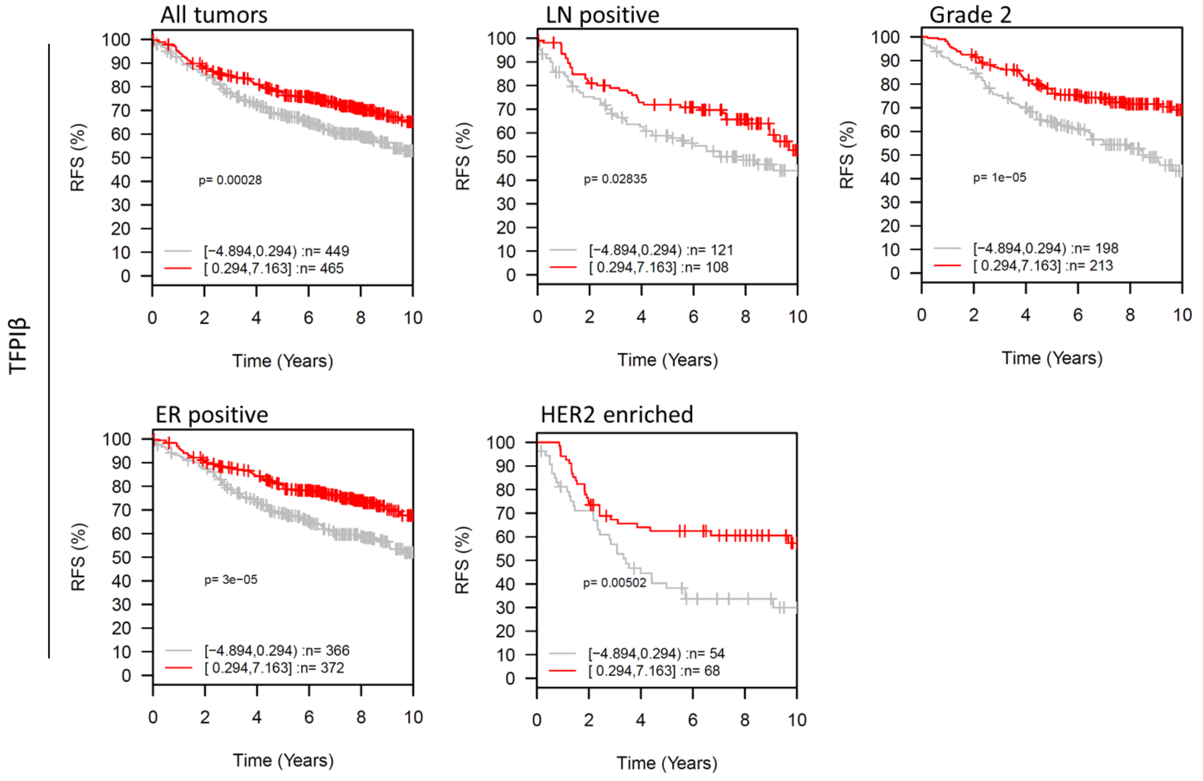

TFPIβ
All tumors
RFS (%)
p= 0.00028
[−4.894,0.294) :n= 449
[0.294,7.163] :n= 465
Time (Years)
LN positive
RFS (%)
p= 0.02835
[−4.894,0.294) :n= 121
[0.294,7.163] :n= 108
Time (Years)
Grade 2
RFS (%)
p= 1e−05
[−4.894,0.294) :n= 198
[0.294,7.163] :n= 213
Time (Years)
ER positive
RFS (%)
p= 3e−05
[−4.894,0.294) :n= 366
[0.294,7.163] :n= 372
Time (Years)
HER2 enriched
RFS (%)
p= 0.00502
[−4.894,0.294) :n= 54
[0.294,7.163] :n= 68
Time (Years)
